# Pressurized Liquid Extraction as a Novel Technique for the Isolation of *Laurus nobilis* L. Leaf Polyphenols

**DOI:** 10.3390/molecules27165099

**Published:** 2022-08-10

**Authors:** Erika Dobroslavić, Ivona Elez Garofulić, Jelena Šeparović, Zoran Zorić, Sandra Pedisić, Verica Dragović-Uzelac

**Affiliations:** Faculty of Food Technology and Biotechnology, University of Zagreb, Pierottijeva 6, 10 000 Zagreb, Croatia

**Keywords:** *Laurus nobilis* L., polyphenols, pressurized liquid extraction (PLE), UPLC-MS/MS, antioxidant activity

## Abstract

*Laurus nobilis* L., known as laurel or bay leaf, is a Mediterranean plant which has been long known for exhibiting various health-beneficial effects that can largely be attributed to the polyphenolic content of the leaves. Pressurized liquid extraction (PLE) is a green extraction technique that enables the efficient isolation of polyphenols from different plant materials. Hence, the aim of this research was to determine optimal conditions for PLE (solvent, temperature, number of extraction cycles and static extraction time) of laurel leaf polyphenols and to assess the polyphenolic profile of the optimal extract by ultra-performance liquid chromatography tandem mass spectrometry (UPLC-MS/MS) as well as to evaluate the antioxidant activity determined by FRAP, DPPH and ORAC assays. The optimal PLE conditions were 50% ethanol, 150 °C, one extraction cycle and 5 min static time. The polyphenolic extract obtained at optimal PLE conditions comprised 29 identified compounds, among which flavonols (rutin and quercetin-3-glucoside) were the most abundant. The results of antioxidant activity assays demonstrated that PLE is an efficient green technique for obtaining polyphenol-rich laurel leaf extracts with relatively high antioxidant activity.

## 1. Introduction

*Laurus nobilis* L., also known as laurel or bay leaf, is an evergreen plant characteristic of the Mediterranean area with high annual rainfall, whose leaves have been widely used as a spice in traditional cuisine, as well as in folk medicine for treating various health conditions. For that reason, their chemical composition and biological activities have been more well researched than other plant parts [1]. Laurel leaves, which have shown antioxidant [2], anti-inflammatory [3] and antimicrobial activity [4], comprise essential oils, alkaloids, polysaccharides, sugars, norisoprenoids, tocopherols, organic acids and a variety of polyphenols comprising flavonoids and non-flavonoids (phenolic acids and lignans) whose structure varies in complexity [5]. Polyphenols can be considered largely responsible for the laurel leaf extracts’ antioxidant activity [2,6] since they possess redox properties which allow them to act as antioxidant agents [7]. Recovery of these antioxidants is a challenging process since the plant material comprises a variety of polyphenolic structures including simple to complex and highly polymerized polyphenols that often interact with other constituents such as polysaccharides and lipids [8]. Therefore, a key step in the utilization of polyphenols’ beneficial properties is establishing an optimal isolation methodology that would result in their effective recovery, and various techniques may be applied for this purpose. Conventional extraction techniques, such as maceration, heat-reflux and infusion, are generally easily applicable, but also often solvent-, time- and energy-consuming. These techniques may also result in the degradation of thermosensitive polyphenolic compounds and are often difficult to automate, making them inapplicable on larger scales. For this reason, many advanced extraction techniques have emerged in recent years. Pressurized liquid extraction (PLE), known as accelerated solvent extraction (ASE), is an automated green extraction technique which was shown to be an economic and time-efficient alternative to conventional techniques since it resulted in comparable or higher contents of polyphenols in various plant extracts [9]. During PLE, the use of elevated pressures allows the liquid solvents to be used at temperatures above their atmospheric boiling point, resulting in enhanced solubility and diffusion rate of the targeted compounds, while the surface tension and solvent viscosity decrease, which results in a drained matrix after the extraction [10]. PLE can be performed in a dynamic or static setup. During the dynamic mode of operation, the solvent is delivered at a constant flow rate, while in the static mode, the extraction process includes one or several cycles in a predetermined time (most often 5–15 min), with solvent replacement between the cycles [10]. Various parameters, such as solvent, temperature, pressure and time of extraction, can be varied in order to improve the extraction performance. After the optimal isolation methodology has been established, the next step is to identify and quantify individual polyphenols and to evaluate the extracts’ antioxidant activity. Combined chromatographic and spectral techniques such as ultra-performance liquid chromatography tandem mass spectrometry (UPLC-MS/MS) are the most useful tools which allow chemical characterization of both simple and complex polyphenolic structures [11]. Several assays divided into two groups (single electron transfer (SET) and hydrogen atom transfer (HAT)) can be used for determination of antioxidant capacity [12]. The ferric reducing antioxidant power (FRAP) and 2,2-di(4-*tert*-octylphenyl)-1-picrylhydrazyl (DPPH) assays are both SET colorimetric assays widely used in estimation of plant extracts’ antioxidant capacities, usually applied together or in a combination with other techniques [13]. Oxygen radical absorbance capacity (ORAC) is a HAT method often chosen for antioxidant capacity determination in plant material since it can measure both polar and nonpolar antioxidants, and it is also the most biologically relevant due to the use of the most biologically prevalent peroxyl radical [14].

Therefore, the purpose of this research was to examine how different PLE parameters (solvent, temperature, number of extraction cycles, static extraction time) affect the phenolic content of laurel leaf extracts and to determine the optimal extraction conditions. To the best of our knowledge, PLE has not been applied for the extraction of laurel leaf polyphenols to date; therefore, there are no data on polyphenolic profiles obtained by this technique. Hence, the goal of this research was to identify and quantify individual polyphenols of the laurel leaf extract obtained by PLE at optimal conditions using the UPLC-MS/MS and to evaluate the antioxidant activity of the extract by using the ORAC, DPPH and FRAP assays.

## 2. Results and Discussion

This study examined the effect of several PLE extraction parameters on the polyphenolic content of laurel leaf extracts. The TPC of the extracts, as determined by the spectrophotometric Folin–Ciocalteu method, is shown in Table 1. The results of the statistical analysis, which was used to determine optimal extraction conditions, are shown in Table 2. Individual compounds in the extract obtained at optimal conditions were identified and quantified by UPLC-MS/MS, while the antioxidant capacity of the optimal PLE extract was characterized by ORAC, DPPH and FRAP assays.

The TPC of the laurel leaf extracts obtained by PLE ranged from 31.87 to 49.30 mg GAE g^−1^, which is similar to the previously reported value of 46.79 mg GAE g^−1^ [15], lower than 59.85 mg GAE g^−1^ [16] and higher than 10.23 mg GAE g^−1^ [17], all obtained by conventional extraction techniques. The TPC of the extracts is also in the range of phenolic contents obtained by microwave-assisted extraction (MAE) (30.88 to 53.57 mg GAE g^−1^) and ultrasound-assisted extraction (UAE) (24.43 to 36.74 mg GAE g^−1^) in our previous research [18].

### 2.1. Pressurized Liquid Extraction (PLE) Optimization

Ethanol percentage in an aqueous solution (50 and 70%), temperature (90, 120 and 150 °C), number of extraction cycles (one, two and three) and static extraction time (5 and 10 min) were varied during the PLE of laurel leaf polyphenols. As shown in Table 2, the ethanol percentage significantly (*p* ≤ 0.05) influenced the TPC of the extracts, which was higher when 50% ethanol was used as a solvent. Similarly, Leyva-Jimenez et al. [19] reported that, in the tested range of 15–85% ethanol, 46% ethanol was optimal for the PLE of polyphenols from *Lippia citriodora* leaves. The use of 50% ethanol also resulted in the highest TPC apple pomace [20] and grape skin extracts [21] obtained by PLE. On the other hand, 71% ethanol was found optimal during PLE of polyphenols from *Myrtus communis* L. leaves [22]. The different observations might be a result of different polyphenolic contents of the samples and the polarity of present compounds which consequently influences the extraction yield when the ‘‘like dissolves like’’ principle is taken into account [23].

Temperature is an important parameter in PLE extraction since it influences the molecular diffusivity and viscosity of the solvent [24]. Generally, applying higher temperatures increases solubility and recovery of compounds from the plant matrix; however, thermosensitive compounds may degrade at higher temperatures, which is why it is crucial to determine optimal temperatures for each plant material [25]. In the present study, the temperature significantly (*p* < 0.01) influenced the TPC of the extracts, which increased proportionally with the increase in temperature. Zhao et al. (2012) [26] have observed a similar effect during the extraction of lignans from *Fructus Schisandrae* where the yield increased proportionally with temperature rise from 80 to 160 °C. A further increase to 180 °C resulted in a lower yield of lignans, possibly due to the mentioned thermal degradation. Repajić et al. have also observed an increase in the TPC of *Urtica dioica* L. leaf extracts [27], as well as *Foeniculum vulgare* Mill. seed extracts [28], with the increase in temperature during PLE. In contrast, temperature had no effect during the PLE of phenolic compounds from *Olea europaea* L. fruit [29].

The number of extraction cycles and the static time are also important since a longer exposure of the analytes to the extraction solvent at elevated temperatures increases the diffusion rate, while multiple extraction cycles may result in the complete extraction of targeted compounds [10]. In the present study, the number of extraction cycles and static extraction time did not significantly influence the TPC of the extracts, contrary to the results reported by Repajić et al. (2020) [27], Li et al. (2019) [21] and Wibisono et al. (2009) [30] where PLE was optimized for the extraction of polyphenols from *Urtica dioica* L. leaves, grape skin and different plant food materials, respectively. For the PLE of phenolic compounds from *Foeniculum vulgare* Mill. seeds, Repajić et al. (2021) [28] reported that static time had no significant influence on the phenolic yield, so 5 min was chosen as optimal, while the number of extraction cycles was a significant parameter. Static time of 5 min was also chosen as optimal for the PLE of *Rosmarinus officinalis* phenolic compounds [31]. Sandei and Vadala (2013) [32] applied one extraction cycle and a static time of 5 min for the optimization of other PLE parameters during the extraction of tomato polyphenols, which are the same as the lowest values applied in our study.

Based on the results of statistical analysis, 50% ethanol, 150 °C, one extraction cycle and a static time of 5 min were chosen as optimal for obtaining the maximum content of polyphenols from laurel leaves.

### 2.2. Polyphenolic Characterization

**Figure 1:**
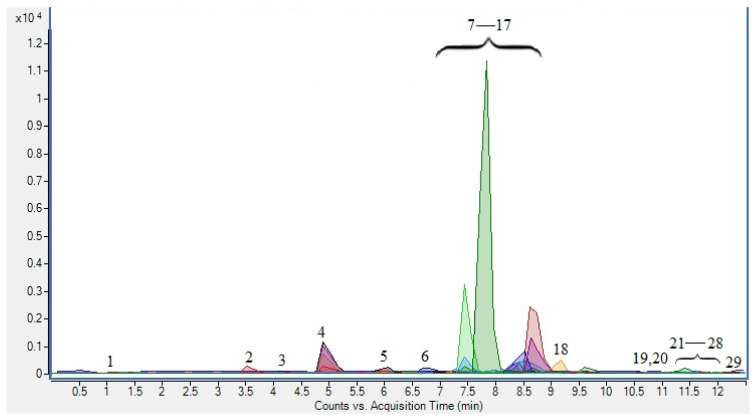
UPLC-MS/MS chromatogram of laurel leaf extracts obtained at optimal PLE conditions in MRM acquisition mode: (1) chlorogenic acid, (2) protocatechuic acid, (3) rosmarinic acid, (4) *p*-coumaric acid, (5) syringic acid, (6) luteolin-6-C-glucoside, (7) rutin, (8) ferulic acid, (9) quercetin-3-glucoside, (10) kaempferol-3-rutinoside, (11) quercetin-3-pentoside, (12) kaempferol-3-*O*-hexoside, (13) luteolin, (14) isorhamnetin-3-hexoside, (15) myricetin, (16) quercetin-3-rhamnoside, (17) epigallocatechin gallate, (18) kaempferol-3-*O*-pentoside, (19) caffeic acid, (20) *p*-hydroxybenzoic acid, (21) apigenin, (22) 3,4-dihidrobenzoic acid hexoside, (23) gallic acid, (24) catechin, (25) epicatechin gallate, (26) procyanidin trimer, (27) apigenin-6-C-(*O*-deoxyhexosyl)-hexoside, (28) epicatechin, (29) kaempferol-3-*O*-deoxyhexoside.

In order to provide insight into the polyphenolic composition of the laurel leaf extract produced under optimal extraction conditions, UPLC-MS/MS analysis was performed (Table 3). Twenty-nine phenolic compounds, including flavonoids (flavones, flavonols, flavan-3-ols and proanthocyanidins) and phenolic acids, were identified in the extract (Figure 1). The identification of compounds was carried out as described in our previous research [18].

**Figure 2:**
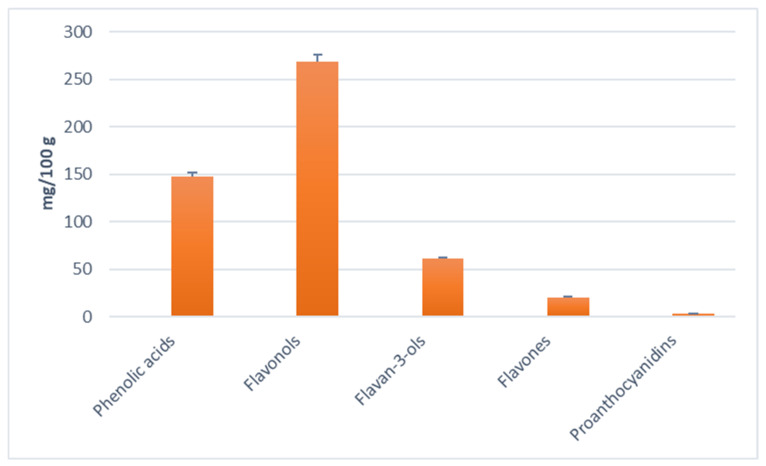
Concentration of different groups of polyphenols determined in the laurel leaf extract obtained at optimal PLE conditions.

According to UPLC-MS/MS results, flavonols were the most abundant group of phenolic compounds (Figure 2), with rutin and quercetin-3-glucoside being the main representatives. Rutin and quercetin were shown to be stable during exposure to elevated temperatures [33], and data on different thermal degradation rates of various quercetin glycosides showed that quercetin-3-*O*-glucoside was among the more stable glycosides [34]. The content of rutin and quercetin-3-glucoside was also the highest in laurel leaf extracts obtained by MAE in our previous research [18]. In the same research, the content of phenolic acids of the extracts obtained by conventional heat-reflux extraction (CRE), MAE and UAE was more than 100 mg g^−1^ lower than that in the extract obtained by PLE in the present study, where caffeic acid was the main representative. Since phenolic acids, especially hydroxycinnamic acids, were shown to be thermally stable [33], the exposure to higher temperatures during PLE may have increased their recovery.

The content of the main flavan-3-ol representatives, catechin and epicatechin, was more than 2-fold lower than their content obtained previously by CRE and more than 3-fold higher than their content obtained previously by UAE and MAE [18]. Even though the high temperature applied during PLE increases the extraction rate, explaining the higher content than in UAE and MAE, thermal degradation of catechin and epicatechin may occur during prolonged exposure to elevated temperatures, leading to their lower content than in the CRE extracts [33,35]. Apigenin was the most abundant flavone in the PLE extract, with a concentration higher than that in extracts obtained by all three techniques applied in the previous research where luteolin was the most abundant flavone [18]. This is in agreement with the findings that apigenin is resistant to prolonged exposure to temperatures around 100 °C, resulting in its higher recovery [36]. Luteolin and its glycosides were previously shown to be less thermally stable than apigenin [33], which is consistent with their recovered amount. The content of procyanidin trimer in the PLE extract was lower than the content in MAE, CRE and UAE extracts obtained previously [18], which is in agreement with previous findings where proanthocyanidins from blueberry and grape pomace were shown to be thermosensitive [37]. In addition, procyanidin oligomers, especially B-type procyanidins, such as the procyanidin trimer detected in the present study, were shown to be the most thermosensitive phenolic compounds in cloudy apple juice [34].

The results of Folin–Ciocalteu spectrophotometry showed significantly higher TPC than that revealed by UPLC-MS/MS, which might be a result of interference caused by non-phenolic constituents present in laurel leaves, such as organic acids, various polysaccharides and sugars [5] which were shown to be detectable by the spectrophotometer [12]. Moreover, it was reported that chlorophyll may interact with the Folin–Ciocalteu reagent, resulting in a seeming increase in the TPC of chlorophyll-rich plant material [38], which could serve as another explanation for the observed discrepancies between the results obtained by chromatographic and spectrophotometric techniques.

### 2.3. Antioxidant Activity

Antioxidant activity of the extract obtained at the defined optimal extraction conditions was determined by the ORAC, DPPH and FRAP assays, and the results are shown in Table 4.

The ORAC value of the laurel leaf extract obtained by PLE was in the range of those reported in the literature, which have varied from 37.7 μmol TE g^−1^ [39] to 170 μmol TE g^−1^ [40] in laurel leaf extracts with lower TPCs (4.04 and 17.66 mg GAE g^−1^, respectively) than in the present study, to even 2600 μmol TE g^−1^ [41] for an extract with a TPC similar to that of the present study (44.07 mg GAE g^−1^). The ORAC value was also in the range of those reported in our previous research carried out on the same laurel leaf sample where CRE resulted in a higher ORAC value (100.09 μmol TE g^−1^) and MAE and UAE resulted in lower ORAC values (86.04 and 90.27 μmol TE g^−1^, respectively), which might have been influenced by the previously discussed differences in the individual phenolic compounds’ contents since the antioxidant activity is dependent on the structural features of phenolic compounds [42]. The DPPH value was lower than the 300 μmol TE g^−1^ reported for a laurel leaf extract [43] where the TPC was 1.01 mg GAE g^−1^. The FRAP value was in the range of 278 μmol TE g^−1^ determined in a hydromethanolic laurel leaf extract where the phenolic content was not analyzed [44], and also lower than 504.25 μmol TE g^−1^ [15] where the TPC of laurel leaf extract was 46.79 mg GAE g^−1^. The differences between the antioxidant activity and the phenolic contents of laurel leaf extracts indicate that the antioxidant activity is not influenced only by the TPC. Several other factors, including the plant growth environment, harvest season, storage conditions and different extraction techniques, may have affected the extracts’ contents of nonphenolic antioxidants such as organic acids, tocopherols and terpenoids [5]. In addition, it is possible that antagonistic or synergistic mechanisms occur between certain components of the extracts which cannot be clarified only by the TPC and could possibly be explained by further qualitative research [45]. Moreover, the content of individual phenolic compounds may significantly influence the antioxidant activity of the extracts since their antioxidant activity depends on the structural features [46]. This is supported by the results of the present study since the same ORAC and DPPH values were determined in the extracts with different TPCs, as well as the contents of individual compounds.

## 3. Materials and Methods

### 3.1. Chemicals and Reagents

Ethanol (96%) and methanol (99.8%) were procured from Lach-ner d.o.o. (Neratovice, Czech Republic), and acetonitrile (HPLC grade) was obtained from J.T. Baker Chemicals (Deventer, the Netherlands). Purified distilled water was produced in a Milli-Q water purification system (Millipore, Bedford, MA, USA). Anhydrous sodium carbonate (≥99.5%), anhydrous sodium acetate (≥99%), formic acid (98–100%), FeCl_3_ × 6H_2_O, sodium phosphate (96%) and Folin–Ciocalteu reagent were from Kemika (Zagreb, Croatia); 2,4,6-tri(2-pyridyl)-s-triazine (TPTZ) and 6-hydroxy-2,5,7,8-tetramethylchroman-2-carboxylic acid (Trolox) were from Acros Organics (Thermo Fisher Scientific, Geel, Belgium); fluorescein sodium salt was from Honeywell Research Chemicals (Bucharest, Romania); and hydrochloric acid (37%), glacial acetic acid, 2,20-azobis (2-amidinopropane) hydrochloride (AAPH) and 2,2-diphenyl-1-(2,4,6-trinitrophenyl)hydrazyl (DPPH) were from Sigma-Aldrich (Steinheim, Germany). Authentic standards of myricetin, quercetin-3-glucoside, caffeic, protocatechuic, gallic, ferulic, rosmarinic, syringic, p-coumaric and chlorogenic acids were procured from Sigma-Aldrich (St. Louis, MO, USA). Kaempferol-3-glucoside, catechin, epicatechin gallate, epigallocatechin gallate, apigenin, rutin, luteolin and procyanidin B2 were purchased from Extrasynthese (Genay, France). Apigenin standard was prepared as an ethanol–0.5% *v/v* dimethyl sulfoxide stock solution, while all other standards were dissolved in methanol. Stock solutions were diluted in order to produce working standard solutions at five concentrations.

### 3.2. Plant Material

Dried laurel leaves collected in the region of Rijeka, Croatia, in November 2020 were purchased from Šafram d.o.o (Zagreb, Croatia) and stored at room temperature. Prior to extraction, the leaves were ground in an electric grinder (OmniBlend, Vervita, Croatia) until a coarse powder was obtained. The total solids of the obtained powder (>95%) were analyzed by drying to constant mass at 103 ± 2 °C [47].

### 3.3. Pressurized Liquid Extraction (PLE)

The extractions were performed on a Dionex ASE 350 Accelerated Solvent Extractor (Thermo Fisher Scientific Inc., Sunnyvale, CA, USA) in static mode. One gram of the ground leaves was combined with 2 g of diatomaceous earth and transferred to 34 mL stainless steel cells previously fitted with three cellulose filters. The extraction conditions were varied following the full factorial design (shown in Table 1): ethanol percentage in aqueous solution (50 and 70%), extraction temperature (90, 120 and 150 °C), extraction cycles (1, 2 and 3) and static extraction time (5 and 10 min). The pressure, purge with nitrogen and volume flush were kept constant at 10.34 MPa, 30 s and 50%, respectively. The 250 mL glass vials with Teflon septa used for the collection of the extracts were filtered into volumetric flasks (50 mL) through Whatman No. 40 filter paper (Whatman International Ltd., Kent, UK) and made up to volume with the extraction solvent. The extracts were transferred and stored in plastic Falcon tubes at −18 °C in a nitrogen gas atmosphere until further analysis. All extracts were prepared in duplicate.

### 3.4. Total Phenolic Content (TPC)

The TPC of laurel leaves was determined following a modified methodology previously established by Shortle et al. [48]. First, 100 µL of the extract (extraction solvent for blank), 200 µL Folin–Ciocalteu reagent and 2 mL of distilled water were put into the reaction tube; 1 mL of sodium carbonate solution (20% *w*/*v*) was added into the reaction after 3 min, and the mixture was shaken using Vortex MS2 Minishaker IKA (IKA, Staufen, Germany) at 1800 rpm and incubated at 50 °C in a water bath. The absorbance was read after 25 min on a VWR UV-1600PC Spectrophotometer (VWR, Wayne, PA, USA) at 765 nm. All measurements were carried out in duplicate. Working standard solutions of gallic acid (50–500 mg L^−1^) were used to prepare a standard calibration curve (y = 0.0035x, R^2^ = 0.9995). The calculated TPC was expressed as a mean value of mg gallic acid equivalents (GAE) per g sample ± standard deviation.

### 3.5. UPLC-MS/MS Conditions

Individual polyphenols in the extracts obtained at optimal conditions were identified and quantified using a UPLC-MS/MS system (Agilent series 1290 RRLC instrument) coupled with an Agilent 6430 Triple Quadrupole LC/MS mass spectrometer (Agilent, Santa Clara, CA, USA). The ionization was performed in positive and negative ionization mode by ESI ion source with nitrogen as a desolvation and collision gas. Drying gas temperature was set at 300 °C, flow rate at 11 L h^−1^, nebulizer pressure at 40 psi and capillary voltage at 4/−3.5 kV. The Zorbax Eclipse Plus C18 column from Agilent (100 *×* 2.1 mm; 1.8 µm particle size) was used for separations under the following conditions: injection volume 2.5 µm and column temperature 35 °C. Other parameters including gradient conditions, solvent composition and instrumental limits of detection (LOD) and quantification (LOQ) were previously reported by Elez Garofulić et al. (2018) [49]. Agilent MassHunter Workstation Software (ver. B.04.01) (Agilent, Santa Clara, CA, USA) was used for data processing and instrument control. Identification and quantitative determination were performed as described by Dobroslavić et al. (2021) [18]. The concentrations of the analyzed polyphenols were expressed as mg per 10^2^ g sample (mean value ± standard deviation). The analyses were carried out in duplicate.

### 3.6. Antioxidant Activity

In order to determine the antioxidant activity of the laurel leaf extract obtained at optimal extraction conditions, three assays were applied.

#### 3.6.1. Oxygen Radical Absorbance Capacity (ORAC) Assay

An automated plate reader (BMG LABTECH, Offenburg, Germany) was used to perform the oxygen radical absorbance capacity (ORAC) assay, while the MARS 2.0 software (BMG LABTECH, Offenburg, Germany) was used for data analysis. The assay was performed following a previously reported method [50]. A 240 mM solution of AAPH, a range of Trolox dilutions (3.12–103.99 μM) and 70.3 nM fluorescein solution were prepared with phosphate buffer (pH 7.4). Afterward, a properly diluted sample or Trolox standard was added to 150 µL of fluorescein in a 96-well microplate which was then incubated at 37 °C for 30 min. The first three cycles set the baseline signal, after which the AAPH solution was injected in order to produce the peroxyl radical. The fluorescence intensity (excitation and emission at 485 and 528 nm, respectively) was monitored for 120 min every 90 s. Determinations were carried out in duplicate. The results were expressed as mean value ± standard deviation of μmol Trolox equivalent (TE) per g sample.

#### 3.6.2. DPPH Radical Scavenging Assay

The DPPH radical scavenging assay was performed following the methodology previously described by Brand-Williams et al. (1995) [51], with some modifications. Briefly, 0.75 mL of the extract and 1.5 mL of 0.2 mM methanolic DPPH solution were mixed in a test tube and shaken at 1800 rpm using Vortex MS2 Minishaker IKA (IKA, Staufen, Germany). As a blank, 2.25 mL of methanol was used. The samples were placed in absence of light at room temperature, and the absorbance was measured after 20 min at 517 nm on a VWR UV-1600PC Spectrophotometer (VWR, Wayne, PA, USA). A standard calibration curve (y = −0.008x + 1.3476, R^2^ = 0.9948) was prepared using standard Trolox solutions in a concentration range of 10–150 μM. The results were expressed as mean value ± standard deviation of μmol TE per g sample.

#### 3.6.3. Ferric Reducing Antioxidant Power (FRAP) Assay

The FRAP assay was performed following a methodology previously described by Shortle et al. (2014) [48], with some modifications. Sodium acetate buffer (0.3 M, pH 3.6), 0.01 M TPTZ solution in 0.04 M hydrochloric acid and 20 mM FeCl_3_ × 6H_2_O aqueous solution in a ratio 10:1:1, respectively, were used to prepare the FRAP reagent. The reagent was incubated at 37 °C for 10 min prior to analysis. Afterward, 80 μL of the extract (extraction solvent for blank), 240 μL of distilled water and 2080 μL of FRAP reagent were added into test tubes, shaken at 1800 rpm using Vortex MS2 Minishaker IKA (IKA, Staufen, Germany) and incubated at 37 °C for 5 min. The absorbance was read on a VWR UV-1600PC Spectrophotometer (VWR, Wayne, PA, USA) at 593 nm. A standard calibration curve (y = 0.0013, R^2^ = 0.9995) was prepared using standard Trolox solutions (25–1000 μM). The results were expressed as mean value ± standard deviation of μmol TE per g sample.

### 3.7. Statistical Analysis

Statistica ver. 12.0 (Statsoft Inc., Tulsa, OK, USA) software was used for the statistical analysis of the results. For the determination of optimal extraction conditions, TPC was the dependent variable, while a full factorial design (mixed two- and three-level) comprising 72 trials was applied in order to evaluate the influence of the following independent variables: (a) solvent (50% and 70% ethanol), (b) temperature (90, 120 and 150 °C), (c) extraction cycles (1, 2 and 3) and (d) static extraction time (5 and 10 min). The normality and homoscedasticity of the data were analyzed using the Shapiro–Wilk W test and Levene’s test, respectively. Normally distributed data were analyzed using one-way and multifactorial analysis of variance (ANOVA), while Tukey’s HSD multiple comparison test was used to compare marginal means. Nonparametric tests including Kruskal–Wallis one-way ANOVA and multiple comparison of mean ranks were applied for analysis of the data which were not normally distributed and/or not homoscedastic. All of the tests were significant at *p* ≤ 0.05.

## 4. Conclusions

PLE, as a novel advanced green extraction technique, was optimized for the efficient isolation of polyphenols from laurel leaves. The optimal extraction conditions determined were 50% ethanol, a temperature of 150 °C, one extraction cycle and a static time of 5 min. The polyphenolic profile of the laurel leaf extract obtained by PLE comprised 29 compounds, including flavonoids (flavones, flavonols, flavan-3-ols and proanthocyanidins) and phenolic acids, and quantitative analysis has shown that flavonols (rutin and quercetin glucoside as the main representatives) were the most abundant group. The antioxidant activity assays have demonstrated that PLE yields extracts with relatively high antioxidant activity through a time-, energy- and solvent-efficient automated process, demonstrating its advantages over conventional extraction techniques in terms of scaling-up processes and reducing solvent, time and energy consumption. Therefore, PLE was proven to be an efficient green extraction technique suitable for the isolation of polyphenols from laurel leaves.

## Figures and Tables

**Table 1:** Total phenolic content of laurel leaf extracts obtained by PLE.

Extraction Parameters				TPC mg GAE g^−1^
% EtOH	Temperature (°C)	Extraction Cycles	Static Extraction Time (min)	
50	90	1	5	34.10 ± 2.02
50	90	1	10	33.82 ± 1.87
50	90	2	5	35.38 ± 1.77
50	90	2	10	36.46 ± 0.81
50	90	3	5	35.39 ± 0.71
50	90	3	10	36.46 ± 0.81
50	120	1	5	36.63 ± 1.31
50	120	1	10	44.03 ± 2.02
50	120	2	5	38.93 ± 1.46
50	120	2	10	40.78 ± 1.56
50	120	3	5	39.85 ± 0.50
50	120	3	10	44.60 ± 1.11
50	150	1	5	46.34 ± 1.21
50	150	1	10	44.46 ± 1.82
50	150	2	5	46.09 ± 1.82
50	150	2	10	47.99 ± 1.11
50	150	3	5	45.82 ± 1.67
50	150	3	10	49.30 ± 1.01
70	90	1	5	31.87 ± 1.51
70	90	1	10	32.41 ± 1.87
70	90	2	5	33.25 ± 0.61
70	90	2	10	34.76 ± 1.56
70	90	3	5	35.39 ± 1.51
70	90	3	10	36.24 ± 2.72
70	120	1	5	35.33 ± 2.27
70	120	1	10	36.42 ± 2.57
70	120	2	5	38.19 ± 0.55
70	120	2	10	40.44 ± 1.51
70	120	3	5	37.49 ± 1.97
70	120	3	10	40.12 ± 2.02
70	150	1	5	43.39 ± 0.86
70	150	1	10	42.98 ± 1.92
70	150	2	5	42.46 ± 1.56
70	150	2	10	47.06 ± 1.46
70	150	3	5	40.56 ± 0.55
70	150	3	10	39.73 ± 1.36

TPC = total phenolic content. Results are expressed as mean ± SD.

**Table 2:** Influence of different PLE parameters on total phenolic content of laurel leaf extracts.

N	Source of Variation	TPC (mg GAE g^−1^)
	% EtOH	*p* < 0.05 ^†^
36	50% *w*/*w*	40.91 ± 0.87 ^b^
36	70% *w*/*w*	38.23 ± 0.72 ^a^
	T	*p* < 0.01 ^†^
24	90 °C	34.63 ± 0.39 ^a^
24	120 °C	39.40 ± 0.62 ^b^
24	150 °C	44.68 ± 0.62 ^c^
	Extraction cycles	*p* = 0.37 ^‡^
24	1	38.48 ± 1.10 ^a^
24	2	40.15 ± 1.01 ^a^
24	3	40.08 ± 0.91 ^a^
	Static extraction time	*p* = 0.14 ^‡^
36	5 min	38.69 ± 0.76 ^a^
36	10 min	40.45 ± 0.86 ^a^

TPC = total phenolic content. N = number of trials. Results are expressed as mean ± standard error. Values marked with different letters are statistically different at *p* ≤ 0.05. ^†^ Statistically significant variable at *p* ≤ 0.05. ^‡^ Statistically insignificant variable at *p* ≤ 0.05.

**Table 3:** Mass spectrometric data on laurel leaf extract obtained at optimal PLE conditions.

Compound	Retention Time	Tentative Identification	Concentration mg/100 g
Phenolic acids			
1	1.008	chlorogenic acid *	0.46 ± 0.01
2	3.638	protocatechuic acid *	58.63 ± 1.66
3	4.259	rosmarinic acid *	0.99 ± 0.03
4	4.937	p-coumaric acid *	4.25 ± 0.12
5	5.961	syringic acid *	0.07 ± 0.00
8	7.917	ferulic acid *	1.01 ± 0.03
19	10.788	caffeic acid *	74.44 ± 2.11
20	10.802	p-hydroxybenzoic acid	2.83 ± 0.08
23	11.573	gallic acid *	0.28 ± 0.01
22	11.426	3.4-dihidrobenz-A-hexoside	4.57 ± 0.13
Flavones			
6	6.938	luteolin-6-C-glucoside	3.91 ± 0.11
13	8.678	luteolin *	7.15 ± 0.20
21	11.415	apigenin *	9.40 ± 0.27
27	11.998	apigenin-6-C-(O-deoxyhexosyl)-hexoside	0.27 ± 0.01
Flavonols			
7	7.561	rutin *	97.31 ± 2.75
9	7.969	quercetin-3-glucoside	94.41 ± 2.67
10	8.349	kaempferol-3-rutinoside	6.00 ± 0.17
11	8.39	quercetin-3-pentoside	7.92 ± 0.22
12	8.64	kaempferol-3-*O*-hexoside	18.02 ± 0.51
14	8.747	isorhamnetin-3-hexoside	24.93 ± 0.71
15	8.791	myricetin *	2.25 ± 0.06
16	8.897	quercetin-3-rhamnoside	9.57 ± 0.27
18	9.178	kaempferol-3-*O*-pentoside	8.04 ± 0.23
29	12.299	kaempferol-3-*O*-deoxyhexoside	0.14 ± 0.00
Flavan-3-ols			
17	9.014	epigallocatechin gallate *	0.15 ± 0.00
24	11.658	catechin *	31.35 ± 0.89
25	11.898	epicatechin gallate *	0.34 ± 0.01
28	12.055	epicatechin	29.20 ± 0.83
Proanthocyanidins			
26	11.977	procyanidin trimer	3.89 ± 0.11
Total phenols (mg 10^−2^ g^−1^)	-	-	501.84 ± 2.27

Results are expressed as mean ± standard deviation. * identification was confirmed with authentic standards.

**Table 4:** Antioxidant activity of laurel extracts obtained by PLE determined by various assays.

Assay	μmol TE g^−1^
ORAC	97.27 ± 2.01
DPPH	73.51 ± 0.22
FRAP	311.10 ± 5.67

Results are expressed as mean ± SD.

## Data Availability

Not applicable.

## Sample Availability

Samples of the compounds are not available from the authors.
